# Global trends in emotional distress

**DOI:** 10.1073/pnas.2216207120

**Published:** 2023-03-27

**Authors:** Michael Daly, Lucía Macchia

**Affiliations:** ^a^Department of Psychology, Faculty of Science & Engineering, Maynooth University, Maynooth W23 F2K8, Ireland; ^b^Department of Psychology, School of Health & Psychological Sciences, City University of London, London EC1V 0HB, United Kingdom

**Keywords:** mental health, distress, COVID-19 pandemic, epidemiology, socioeconomic status

## Abstract

In this study, we examined emotional distress using annual representative survey data from 1.53 million individuals surveyed in 113 countries from 2009 to 2021. Participants reported whether they had experienced worry, sadness, stress, or anger during a lot of the previous day. Within-country estimates showed that the prevalence of feelings of emotional distress increased from 25 to 31% between 2009 and 2021, with those with low levels of education and income experiencing the largest increases in distress. On a global level, the pandemic period was characterized by an initial increase in distress in 2020 followed by recovery in 2021.

Emerging evidence suggests that psychological distress has risen substantially in recent years in the United States and the United Kingdom (1–4). Stimulated by evidence documenting rising “deaths of despair” and ill-being in the United States (1), a recent study showed that extreme distress among US adults rose from 3.6% in 1993 to 6.4% in 2019 (2). This rise was linked to low-education and labor market precarity. A second US study (3) found a rise in distress among working-aged adults from 16.1% in 1999–2000 to 22.6% in 2017–2018. In the United Kingdom, increases in distress have been identified since 2010 in young adults and since 2015 in working-age adults (4). Further, there is evidence that these increases may have been exacerbated by the COVID-19 pandemic (5, 6).

On a worldwide scale, the population shocks of the financial crisis in 2008 to 2010 (7) and the COVID-19 pandemic in 2020 (8) have been linked to increased mental health difficulties and emotional distress in affected nations. However, a global picture of contemporary trends in distress has not yet emerged. To address this issue, we examine the most up-to-date Gallup World Poll representative survey data from 113 nations to estimate global changes in feelings of emotional distress from 2009 to 2021, including during the COVID-19 pandemic.

## Results

**Fig. 1.**
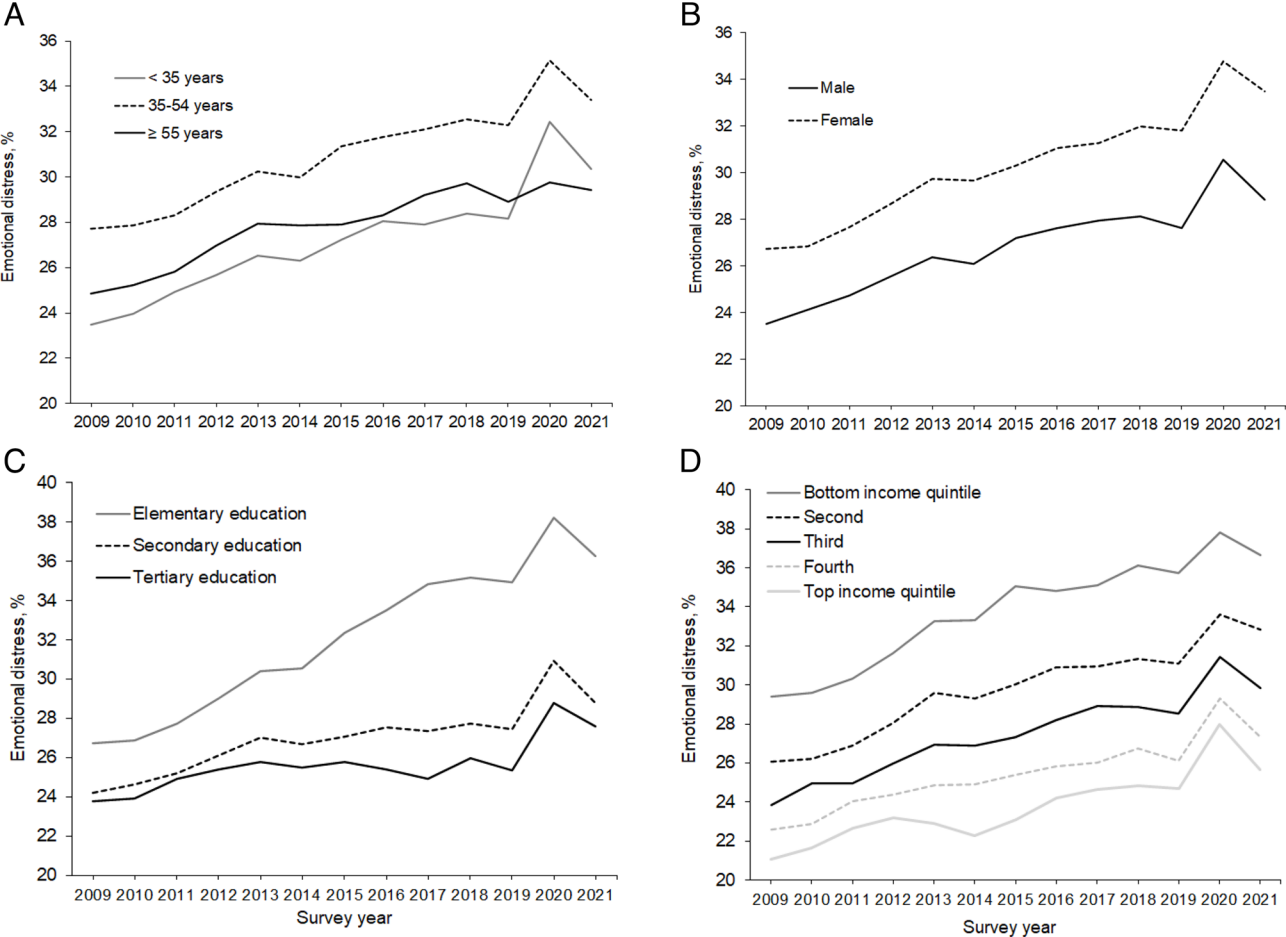
Estimated time trends in emotional distress among 1.53 million participants aged 15 y and older in 113 countries between 2009 and 2021. Figure panels are based on regression analysis and display distress trends by (*A*) age group, (*B*) participant sex, (*C*) education levels, and (*D*) income quintiles.

Regression analyses showed that the prevalence of feelings of distress rose from 25.16% in 2009 to 31.19% in 2021, an overall increase of 6.03 percentage points [95% CI (4.32, 7.75)]. Statistically significant increases in emotional distress levels between 2009 and 2021 were observed across all demographic groups examined (Fig. 1 and Table 1) and were largest among those with elementary-level education [9.53%, 95% CI (7.06, 11.99)] and those in the bottom income quintile [7.27%, 95% CI (5.44, 9.10)]. From 2009 to 2021, there was a substantial increase in feelings of stress [9.97%, 95% CI (7.38, 12.56)], sadness [6.31%, 95% CI (4.41, 8.22)], and worry [6.22%, 95% CI (4.00, 8.47)]. Anger did not increase significantly over the study period [1.61%, 95% CI (−0.1, 3.32)].

**Table 1.** Estimated changes in emotional distress between 2009 and 2021 by population subgroups

Variable	Distress level 2009 % (95% CI)	Distress level 2021 % (95% CI)	Change in distress 2009 to 2021^*^ % (95% CI)
Age group			
Aged under 35 y	23.48 (22.30, 24.68)	30.36 (29.23, 31.48)	6.87 (5.06, 8.67)
Aged 35 to 54 y	27.72 (26.43, 29.02)	33.38 (32.28, 34.48)	5.56 (3.71, 7.61)
Aged ≥55 y	24.86 (23.48, 26.24)	29.44 (28.22, 30.65)	4.57 (2.63, 6.52)
Sex			
Male	23.53 (22.38, 24.68)	28.84 (27.91, 29.78)	5.31 (3.65, 6.98)
Female	26.73 (25.50, 27.96)	33.48 (32.15, 34.80)	6.75 (4.74, 8.76)
Level of education			
Elementary	26.73 (25.08, 28.38)	36.25 (34.78, 37.73)	9.53 (7.06, 11.99)
Secondary	24.18 (23.05, 25.32)	28.79 (27.89, 29.69)	4.61 (3.03, 6.19)
Tertiary	23.79 (22.22, 25.35)	27.58 (26.59, 28.58)	3.80 (1.74, 5.85)
Income quintile			
Bottom	29.40 (28.13, 30.67)	36.68 (35.42, 37.94)	7.27 (5.44, 9.10)
Second	26.09 (24.67, 27.51)	32.83 (31.54, 34.13)	6.75 (4.55, 8.94)
Third	23.83 (22.58, 25.07)	29.85 (28.73, 30.96)	6.02 (4.09, 7.95)
Fourth	22.58 (21.30, 23.85)	27.31 (26.23, 28.38)	4.73 (2.89, 6.56)
Top	21.08 (19.83, 22.33)	25.64 (24.64, 26.64)	4.56 (2.82, 6.30)

Note. Estimates are based on ordinary least squares regression analyses including survey year as a categorical variable and including country fixed-effects. Sampling weights are applied; 95% CIs not including zero are statistically significant at the *P* < 0.05 level.

^*^Coefficients are estimated using a categorical survey year variable entered into an ordinary least squares regression model and indicate the change in distress levels from 2009 to 2021.

The 2020 pandemic dummy variable was statistically significant [B = 2.49, 95% CI (0.71, 4.27)] in a model accounting for the curvilinear time trend in distress, indicating that in 2020, distress rose by 2.5 percentage points over and above the existing prepandemic trend in distress. Significant increases in distress in 2020 were found among most demographic groups except those aged ≥55 y and the lowest education and income groups. The largest increase in distress observed in 2020 was among those aged under 35 y [B = 3.98, 95% CI (2.02, 5.93)]. Distress levels declined from 2020 to 2021 [B = −1.47, 95% CI (−2.55, −0.39)]. The dummy variable for the 2021 wave was not statistically significant [B = 1.04, 95% CI (−0.74, 2.82)]. This indicated that by 2021, distress levels did not deviate significantly from the existing time trend in distress, as estimated using prepandemic distress data.

## Discussion

Using a 113-country sample of over 1.5 million adults, this study provides insight into recent global changes in emotional distress. From 2009 to 2021, the prevalence of feelings of distress increased markedly from 25 to 31 percent, an increase of 6 percentage points or 24 percent. Increases in distress were found across demographic groups and were largest among more disadvantaged groups. These findings are consistent with evidence of rising distress (3) and growing socioeconomic disparities in distress in the United States (2). Understanding the factors (e.g., economic insecurity, political instability, reduced social cohesion) that account for potential widening disparities in distress on a global scale will now be crucial. It will also be important to ascertain the health implications of rising distress levels, including for distress-related outcomes such as rising opioid use (1–3, 9, 10).

We found that distress levels rose by 2.5 percentage points during the pandemic in 2020 over and above the general increasing time trend in distress. This finding is in line with evidence from longitudinal studies indicating that the pandemic had an adverse psychological effect which was small in magnitude (8). Further, the pandemic-related increase in distress found in this study was short lived. Distress levels declined from 2020 to 2021 and at this point were not greater than expected based on prepandemic trends. This result is consistent with findings suggesting that populations flexibly adapted to the stressful circumstances of the pandemic and recovered relatively quickly from the distressing impact of the initial lockdown period (5, 8, 11).

This study draws on global survey data to quantify recent population-level changes in emotional distress including during the COVID-19 pandemic. However, the study is limited in its reliance on a brief self-reported measure of distress, relatively small national annual samples, which can increase sampling error, and reliance on a subset of 113 countries to estimate global changes in emotional distress.

## Materials and Methods

### Sample.

Gallup surveyed 1.76 million individuals from 165 countries from 2009 to 2021. The target population for the Gallup World Poll (GWP) is the noninstitutionalized civilian population of the world, aged 15 y and older (12). Random-digit-dialing of a nationally representative list of telephone numbers was used to conduct telephone surveys in countries where telephone coverage represents at least 80% of the population. Surveys were administered in person in regions with less extensive telephone coverage, including most of Asia, the Middle East, and Africa. During the pandemic phone-interviews were conducted in most nations. In all countries, households were randomly selected, and approximately 1,000 individuals were surveyed each year. Nations were included in our analyses if data were available for more than half of the survey waves between 2009 and 2021, including during the pandemic. This produced a sample size of 1,527,616 individuals from 113 countries. (Countries included are listed in *SI Appendix*.) On average, the countries included completed 10 of 13 survey waves. Details of the sampling frame and training and field quality control procedures can be found in the Gallup Worldwide Research Methodology and Codebook (12). This study involved secondary analysis of the GWP anonymized microdata files, which did not require institutional approval from the Maynooth University Social Research Ethics Sub-Committee.

### Emotional Distress.

Distress was assessed using four items that asked whether participants experienced different negative feelings the previous day: stress, worry, sadness, and anger. These items align with the most prominent components of emotional distress typically assessed, which include anxiety, depression, and anger (13). Participants were first asked to “please think about yesterday, from the morning until the end of the day. Think about where you were, what you were doing, who you were with, and how you felt.” Participants were then asked, “Did you experience the following feelings during A LOT OF THE DAY yesterday?” and they indicated whether they experienced each emotion (coded as 1 = yes and 0 = no). Scores were scaled as percent of maximum possible (POMP) scores to produce a score indicating the percentage of distressing feelings experienced (ranging from 0 to 100%). POMP scoring was used to allow regression coefficients to be reported in percentage point terms and to facilitate comparisons between total distress scores and individual distress item scores. The measure had an internal reliability of α = 0.69 and an average interitem correlation of 0.37 (range 0.29 to 0.44), indicating satisfactory internal consistency (14).

### Demographics.

Participants reported their age in years (grouped under 35, 35 to 54, and 55+ y), their sex (male and female), highest level of education (elementary, secondary, and tertiary level), and income levels (grouped into country-specific income quintiles).

### Statistical Analysis.

Ordinary least squares regression was used to estimate changes in emotional distress levels from 2009 to 2021 for the overall sample and age, sex, education, and income groups. To estimate the deviation of distress from expected levels during the pandemic, we estimated a model including linear and quadratic time terms for the number of years since 2009. A pandemic dummy variable (0 = 2009 to 2019, 1 = 2020) was then added to this model to capture the extent to which distress levels in 2020 deviated from the general time trend for this measure. We repeated this analysis including a dummy variable for 2021 (0 = 2009 to 2019, 1 = 2021) to test whether distress levels in 2021 deviated from the overall pattern of change in distress. This approach has been used to test whether the magnitude of changes in mental health observed during the pandemic significantly exceeds the changes that would be expected based on existing trends in mental health in the years prior to the pandemic (15).

SEs were clustered at the country level to account for correlations in distress scores among those sampled from each nation. Country fixed-effects were included in all models to ensure that within-country changes in distress were estimated. Sampling weights were applied to adjust for oversampling, the differential probability of selection into the sample based on household size, and to provide a poststratification adjustment based on population demographics where available. Each country was weighted equally in our analyses.

## Supplementary Material

Appendix 01 (PDF)

## Data Availability

The Gallup World Poll data belong to Gallup, Inc. For more information, see: https://www.gallup.com/analytics/318875/global-research.aspx (16). Scripts for analyses are available through the Open Science Framework (OSF), https://osf.io/yc9j4/?view_only=9459b20e6d3d4800af11cea55f9fab11 (17).
