# Supplementary material for: Global trends in emotional distress
Source: Proc Natl Acad Sci U S A. 2023 Mar 27;120(14):e2216207120. doi: 10.1073/pnas.2216207120 (PMC10083620; doi:10.1073/pnas.2216207120)
Supplement: Supplementary file 1 — Appendix 01 (PDF) [file pnas.2216207120.sapp.pdf]

SUPPLEMENTAL INFORMATION

Global Trends in Emotional Distress

Countries Sampled by World Region:

Western Europe (N = 18 countries): Austria, Belgium, Cyprus, Denmark, Finland, France, Germany, Greece, Ireland, Italy, Malta, Netherlands, Norway, Portugal, Spain, Sweden, Switzerland, United Kingdom.

Central and Eastern Europe (N = 16 countries): Albania, Bosnia and Herzegovina, Bulgaria, Croatia, Czech Republic, Estonia, Hungary, Kosovo, Latvia, Lithuania, Montenegro, North Macedonia, Poland, Romania, Serbia, Republic of Slovenia.

Commonwealth of Independent States (N = 9 countries): Armenia, Georgia, Kazakhstan, Kyrgyzstan, Moldova, Russia, Tajikistan, Ukraine, Uzbekistan.

Middle East and North Africa (N = 13 countries): Algeria, Bahrain, Egypt, Iran, Iraq, Israel, Jordan, Lebanon, Morocco, Saudi Arabia, Tunisia, Turkey, United Arab Emirates.

Sub-Saharan Africa (N = 19 countries): Benin, Burkina Faso, Cameroon, Cote d'Ivoire, Ethiopia, Gabon, Ghana, Guinea, Kenya, Malawi, Mali, Mauritius, Nigeria, Senegal, South Africa, Tanzania, Togo, Uganda, Zambia.

North America and Australia & New Zealand (N = 4 countries): Australia, Canada, New Zealand, United States.

Latin America & Caribbean (N = 16 countries): Argentina, Bolivia, Brazil, Chile, Colombia, Costa Rica, Dominican Republic, Ecuador, El Salvador, Honduras, Mexico, Nicaragua, Panama, Paraguay, Peru, Uruguay.

South Asia (N = 5 countries): Afghanistan, Bangladesh, India, Pakistan, Sri Lanka.

Eastern Asia (N = 5 countries): China, Japan, Mongolia, South Korea, Taiwan.

Southeast Asia (N = 8 countries): Cambodia, Indonesia, Malaysia, Myanmar, Philippines, Singapore, Thailand, Vietnam.
